# Unraveling the Complexity of the Cancer Microenvironment With Multidimensional Genomic and Cytometric Technologies

**DOI:** 10.3389/fonc.2020.01254

**Published:** 2020-07-23

**Authors:** Natasja L. de Vries, Ahmed Mahfouz, Frits Koning, Noel F. C. C. de Miranda

**Affiliations:** ^1^Pathology, Leiden University Medical Center, Leiden, Netherlands; ^2^Immunohematology and Blood Transfusion, Leiden University Medical Center, Leiden, Netherlands; ^3^Human Genetics, Leiden University Medical Center, Leiden, Netherlands; ^4^Delft Bioinformatics Laboratory, Delft University of Technology, Delft, Netherlands; ^5^Leiden Computational Biology Center, Leiden University Medical Center, Leiden, Netherlands

**Keywords:** cancer microenvironment, single-cell, data integration, multi-omics, mass cytometry, spatial analysis, immunophenotyping

## Abstract

Cancers are characterized by extensive heterogeneity that occurs intratumorally, between lesions, and across patients. To study cancer as a complex biological system, multidimensional analyses of the tumor microenvironment are paramount. Single-cell technologies such as flow cytometry, mass cytometry, or single-cell RNA-sequencing have revolutionized our ability to characterize individual cells in great detail and, with that, shed light on the complexity of cancer microenvironments. However, a key limitation of these single-cell technologies is the lack of information on spatial context and multicellular interactions. Investigating spatial contexts of cells requires the incorporation of tissue-based techniques such as multiparameter immunofluorescence, imaging mass cytometry, or *in situ* detection of transcripts. In this Review, we describe the rise of multidimensional single-cell technologies and provide an overview of their strengths and weaknesses. In addition, we discuss the integration of transcriptomic, genomic, epigenomic, proteomic, and spatially-resolved data in the context of human cancers. Lastly, we will deliberate on how the integration of multi-omics data will help to shed light on the complex role of cell types present within the human tumor microenvironment, and how such system-wide approaches may pave the way toward more effective therapies for the treatment of cancer.

## Introduction – Heterogeneity of Cancer and Need for Multidimensional Approaches

A genetic basis for cancer development was first proposed by the German zoologist Theodor Boveri who speculated that malignant tumors might be the result of abnormal chromosome alterations in cells (1). By then, a cancer cell-centric vision dominated, where tumorigenesis was thought to be exclusively driven by multistep alterations in cellular genomes. During the last decades, however, it has become increasingly apparent that the study of cancers must also encompass other constituents of the cancer microenvironment including immune cells, fibroblasts, and other stromal components, to capture all aspects of cancer biology (2). The immune system, for example, plays a dichotomous role in cancer development and progression, as different cells can antagonize or promote tumorigenesis (3). The mapping and understanding of the interplay between cancer cells and other constituents of the cancer microenvironment is thus fundamental for the clinical management of this disease.

The study of cancers as complex systems is further complicated by cancer heterogeneity that can occur at different levels; intratumorally, between lesions, and across patients. Intratumoral heterogeneity involves the near-stochastic generation of both genetic (e.g., mutations, chromosomal aberrations) and epigenetic (e.g., DNA methylation, chromatin remodeling, post-translational modification of histones) modifications. Within tumors, distinct niches can favor the outgrowth of different cancer cell clones that acquired characteristics compatible with regional microenvironments (e.g., nutrient and oxygen availability, exposure to immune cells). Other intrinsic sources of heterogeneity such as self-renewal of cancer cells and cell differentiation processes contribute further to intratumoral heterogeneity (4, 5). In addition, the immune system is a major part of the tumor microenvironment and contains many different types of adaptive (e.g., CD4^+^ and CD8^+^ T lymphocytes) and innate (e.g., macrophages and innate lymphoid cells) immune cells that also contribute to cancer heterogeneity (6). Their location within a tumor has been shown to significantly impact their anti- or pro-tumorigenic effects (7). In addition, the density of immune cell infiltration in tumors is a well-known determinant for the prognosis of cancer patients (8). Inter-lesional heterogeneity can be observed between multiple primary tumors at time of diagnosis, between a primary tumor and metastasis, and between different metastatic niches in individual patients. They can be a result of the outgrowth of subclones that can be (epi)genetically distinguished by mutations or structural variations (9). Moreover, the structure and composition of the cancer microenvironment can vary between the primary tumor and metastases. Upon extravasation, cancer cells from primary tumors are exposed to different types of immune cells, stromal cells, platelets, and metabolic stress, and have to adapt to the new tissue microenvironment. As such, the metastatic tissue (“soil”) plays a critical role in regulating the growth of metastases (“seed”) (10). Finally, interpatient heterogeneity is, on top of the aforementioned variables, also fueled by distinct germline genetic backgrounds and environmental and stochastic factors that can affect cancer progression but also immunity.

**Figure 1:**
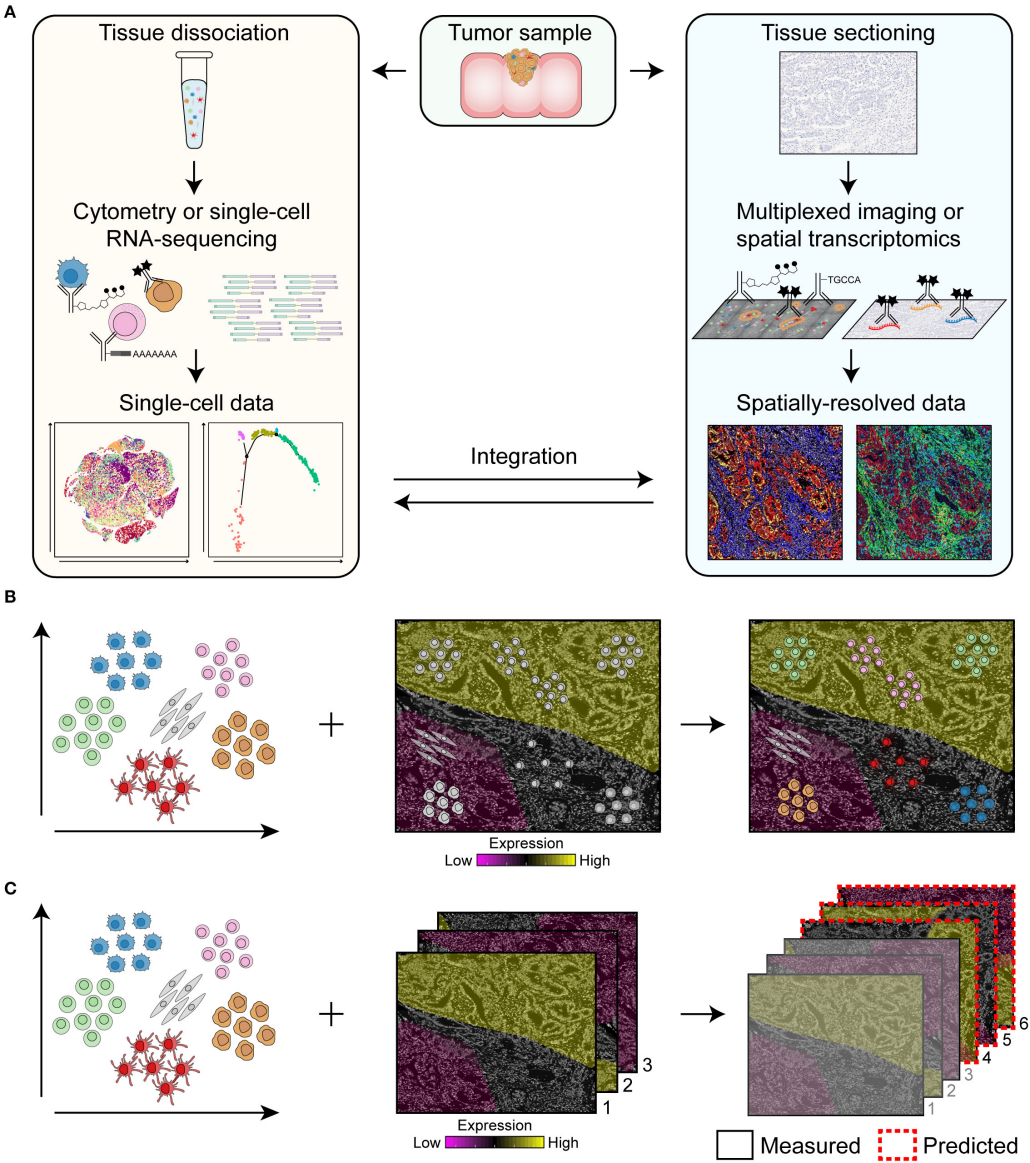
Overview of the pipeline for the integration of single-cell data of dissociated cells and spatially-resolved data. **(A)** Single-cell data can be obtained by flow and mass cytometry that make use of antibodies coupled to fluorochromes or heavy metal isotopes, respectively, for the immunodetection of dissociated cells. For single-cell RNA-sequencing, antibodies coupled to oligonucleotides can be used to simultaneously retrieve information on protein and RNA expression of single cells. Spatially-resolved data can be obtained by multiplexed imaging or spatial transcriptomics by immunodetection of tissue sections with antibodies coupled to fluorochromes, heavy metal isotopes or oligonucleotides. Integration of single-cell data of dissociated cells with spatially-resolved data will reveal the full cellular landscape of the cancer microenvironment. **(B,C)** Integration approaches for single-cell data of dissociated cells and spatially-resolved data. Single-cell data of dissociated cells can be used as guide for cell type identification in spatial data and, vice versa, spatially-resolved data can be used to predict the location of dissociated cells based on the similarity of their expression profiles to spatially-mapped data **(B)**. In addition, single-cell data can be used to predict the spatial profile of genes or proteins in the samples that have not been measured to expand the coverage of spatial data **(C)**. Based on samples that have been measured (i.e., sample 1, 2, and 3), the expression of genes or proteins in sample 4, 5, and 6 can be predicted.

Major challenges in the field of cancer research are the identification of predictive biomarkers to select patients that are likely to respond to specific treatments, the detection of mechanisms of resistance to therapy, and the development of novel treatments to improve cancer survival. Here, we review the rise of cutting-edge multidimensional technologies such as spectral flow cytometry, multiparameter immunofluorescence, (imaging) mass cytometry, single-cell RNA-sequencing (scRNA-seq), and RNA spatial profiling that may play a crucial role to address the former problems. We will discuss how multi-omics of dissociated cells as well as of spatial data can be obtained (Figure 1A) and the importance of integrating them to reveal the full cellular landscape of the cancer microenvironment (Figures 1B,C). For example, single-cell data of dissociated cells can be used as guide for cell type identification in spatial data (11) and, vice versa, spatial data can be used to predict the location of dissociated cells based on the similarity of their expression profiles to spatially-mapped data (12–14) (Figure 1B). In addition, mapping can be used to predict the spatial profile of genes or proteins which have not been experimentally measured to expand the coverage of spatial data (Figure 1C) (15–17).

## Multidimensional Single-Cell Technologies and Their Strengths and Weaknesses

### Single-Cell DNA- and RNA-Sequencing

Next-generation sequencing (NGS) approaches have revolutionized our ability to generate high-throughput genomic data where individual RNA and DNA molecules are represented by sequencing reads thereby retaining information on genotypes, phenotypes, cellular states, and sub-clonal alterations. Traditional molecular profiling has, until recently, largely relied on the analysis of bulk cell populations. Deep sequencing of DNA and RNA from tissues enabled reconstruction of “average” genomes and “average” transcriptomes that could then be deconstructed by employing bioinformatic algorithms to perform clonal evolution analysis or determine the composition of cancer microenvironments (18–21). For an unbiased and systematic characterization of cells, high-throughput single-cell DNA- and RNA-sequencing have emerged as powerful tools. With single-cell DNA-sequencing, the genomic heterogeneity of tissues can be explored in detail. It can be used to detect nucleotide variations and chromosomal copy number alterations as well as more complex genomic rearrangements and cellular fractions. Single-cell genome sequencing involves whole-genome amplification of single cells, of which the three main methods are MDA (22), MALBAC (23), and DOP-PCR (24). In 2011, the first study of DNA-sequencing of human breast cancer single cells was published (25), which was followed by many single-cell studies charting genetic heterogeneity within individual tumors as well as between primary tumors and their metastases, thereby allowing for a detailed understanding of the evolution processes occurring in a tumor. Single-cell DNA-sequencing has myriad applications in cancer research including examining intratumoral heterogeneity (26–28), investigating clonal evolution during tumorigenic processes (25, 29–32), tracing metastatic dissemination (33), genomic profiling of circulating tumor cells (34–36), measuring mutation rates (37), and gain insight into resistance to therapy (38). By defining, in detail, the genetic composition of tumors, the rationalization of targeted cancer therapies is made possible. However, drawbacks of single-cell DNA-sequencing methods are non-uniform coverage and allelic dropout events as well as artifacts introduced during genomic amplification, all of which contribute to a high rate of false negative and false positive findings (39).

The first single-cell RNA-sequencing (scRNA-seq) experiment was published in 2009 by Tang and colleagues who profiled the transcriptome of a single cell from early embryonic development (40). Rapid technological advances resulted in an exponential increase in the number of cells that can be studied by scRNA-seq analyses (41). Just 8 years later, 10x Genomics published a scRNA-seq dataset of more than one million individual cells from embryonic mice brains (42). There are many different scRNA-seq library preparation platforms, which can be categorized into plate-based, droplet-based, and microwell-based (41). The selection of the method depends on the research question, the number of input cells, the sequencing depth, the need for full-length coverage of transcriptomes, and costs, among others [reviewed by (43, 44)]. ScRNA-seq has demonstrated to be a powerful technique to decipher cancer biology. In 2012, Ramskold et al. applied scRNA-seq to study circulating tumor cells in melanoma, and could identify potential biomarkers for melanoma as well as SNPs and mutations in this relatively rare circulating tumor cell population (45). Thereafter, scRNA-seq has been used to study the microenvironment of several cancer types including prostate cancer (46), breast cancer (47), glioma (48–50), renal cancer (51), lung cancer (52), melanoma (53–56), colorectal cancer (57–59), pancreatic ductal adenocarcinoma (60), liver cancer (61), head and neck cancer (62), leukemia (63), and glioma (64). A pioneering study that applied scRNA-seq to primary glioblastomas uncovered inherent variability in oncogenic signaling, proliferation, immune responses, and regulators of stemness across cells sorted from five tumors (48). However, this study was restricted to cancer cells and did not further investigate other cell types of the cancer microenvironment. Subsequently, another scRNA-seq study examined distinct genotypic and phenotypic states of malignant, immune, stromal, and endothelial cells of melanomas from 19 patients (53). They identified cell states linked to resistance to targeted therapy, interactions between stromal factors and immune cell abundance, and potential biomarkers for distinguishing dysfunctional and cytotoxic T cells. A recent study in colorectal cancer broadened such scRNA-seq analysis by including a comparison of primary tumors to matched normal mucosa samples (58). By projecting their scRNA-seq data to a large reference panel, the authors identified distinct subtypes of cancer-associated fibroblasts and new expression signatures that were predictive of prognosis in colorectal cancer. Further, scRNA-seq has been applied to investigate changes in the tumor microenvironment of cancer patients treated with immune checkpoint blockade to find signatures associated with positive responses to this therapy (65, 66).

Currently scRNA-seq can be combined with sequencing of T cell receptor and immunoglobulin repertoires thereby allowing to connect information of B- and T cell specificity and phenotype. High-throughput single-cell B cell receptor sequencing of more than 250,000 B cells from different species has recently been pioneered to obtain paired antibody heavy- and light chain information at the single-cell level, and revealed a rapid discovery of antigen-reactive antibody candidates (67). By a novel approach called RAGE-seq (Repertoire and Gene Expression by Sequencing), gene expression profiles can be paired with targeted full-length mRNA transcripts providing BCR and TCR sequences (68). This method has been applied to study cells from the primary tumor and tumor-associated lymph node of a breast cancer patient and demonstrated the ability to track clonally related lymphocytes across tissues and link TCR and BCR clonotypes with gene expression features (68). A limitation of scRNA-seq is that RNA levels are not fully representative of protein amounts. The advent of CITE-seq, REAP-seq, and Abseq overcame this limitation by enabling simultaneous detection of gene expression and protein levels in single cells by combining oligonucleotide-labeled antibodies against cell surface proteins with transcriptome profiling of thousands of single cells in parallel (69–71). scRNA-seq, when employed in a discovery setting, can inform on the best markers to be used for the study of specific populations by complementary technologies such as flow or mass cytometry. However, aspects of sample preparation and handling have been shown to induce significant alterations in the transcriptome (72). Furthermore, throughput is limited by cost, protocol complexity, available sequencing depth, and dropout events. Together, this can affect the downstream analysis pipeline such as clustering of cell populations and the inference of cellular relationships.

Computational analysis of scRNA-seq data is challenging and involves multiple steps, e.g., quality control, normalization, clustering, and identification of differentially expressed genes and/or trajectory inferences. Multiple unsupervised clustering analyses are available to identify putative cell types, of which graph-based clustering is most widely used (73). For each of these steps, numerous computational tools are available, but in addition software packages have implemented the entire clustering workflow such as Seurat (16), scanpy (74), and SINCERA (75).

### Single-Cell Epigenetic Characterization

Although most high-throughput profiling studies to date have focused on DNA, RNA, and protein expression, recent progress in studying the epigenetic regulation of gene expression, at single-cell level, has been made. Over the last decades, methods have been developed including ATAC-seq to measure chromatin accessibility (76), bisulfite sequencing to measure DNA methylation (77), ChIC-sequencing to measure histone modifications (78), and chromosome conformation capture (3C) to analyze the spatial organization of chromatin in a cell (79). Several studies revealed epigenetic programs that regulate T cell differentiation and dysfunction in tumors. Analysis of chromatin accessibility by ATAC-seq revealed that CD8^+^ T cell dysfunction is accompanied with a broad remodeling of the enhancer landscape and transcription factor binding as compared to functional CD8^+^ T cells in tumors (80–83). Also, an increased chromatin accessibility at the enhancer site of the *PDCD1* gene (encoding for PD-1) has been found in the context of dysfunctional CD8^+^ T cells (82). In addition, studies have applied epigenetics to determine mechanisms of resistance to cancer immunotherapies by characterizing chromatin regulators of intratumoral T cell dysfunction before and after PD-1, PD-L1, or CTLA-4 blockade therapy (84, 85). Lastly, DNA hypermethylation may result in the inactivation of genes, such as mismatch repair gene *MLH1* associated with microsatellite instability in colorectal cancer (86). Until recently, studies on epigenetic modifications depended on correlations between bulk cell populations. Since 2013, with the development of single-cell technologies, epigenomic techniques have been modified for application to single cells to study cell-to-cell variability in for instance chromatin organization in hundreds or thousands of single cells simultaneously. Several single-cell epigenomic techniques have been reported on recently, including measurements of DNA methylation patterns (scRRBS, scBS-seq, scWHBS) (87–89), chromatin accessibility (scATAC-seq) (90), chromosomal conformations (scHi-C) (91), and histone modifications (scChIC-seq) (92). A recent study applied scATAC-seq to characterize chromatin profiles of more than 200,000 single cells in peripheral blood and basal cell carcinoma. By analyzing tumor biopsies before and after PD-1 blockade therapy, Satpathy et al. could identify chromatin regulators of therapy-responsive T cell subsets at the level of individual genes and regulatory DNA elements in single cells (93). Interestingly, variability in histone modification patterns in single cells have also been studied by mass cytometry, which was denominated EpiTOF (94). In this way, Cheung et al. identified a variety of different cell-type and lineage-specific profiles of chromatin marks that could predict the identity of immune cells in humans. Lastly, scATAC-seq has been combined with scRNA-seq and CITE-seq analyses to find distinct and shared molecular mechanisms of leukemia (95). These single-cell strategies will allow to further understand how the epigenome drives differentiation at the single-cell level and unravel drivers of epigenetic states that could be used as target for the treatment of cancer. Additionally, these methods may be used to measure genome structure in single cells to define the 3D structure of the genome. However, for many of these single-cell epigenetic techniques, disadvantages are the low coverage of regulatory regions such as enhancers (scRRBS), low coverage of sequencing reads (scChiP-seq, scATAC-seq), and low sequencing resolution (scHi-C) (96, 97).

### Single-Cell Protein Measurements

Flow cytometry has been, in the past decades, the method of choice for high-throughput analysis of protein expression in single cells. The number of markers that can be simultaneously assayed was limited to ~14 markers due to the broad emission spectra of the fluorescent dyes. Recent developments with spectral flow cytometer machines enable the detection of up to 34 markers in a single experiment by measuring the full spectra from each cell, which are unmixed by reference spectra of the fluorescent dyes and the autofluorescence spectrum (98). Fluorescence emission is registered by detectors consisting of avalanche photodiodes instead of photomultiplier tubes used in conventional flow cytometry. A variety of cellular features can be detected by flow cytometry including DNA and RNA content, cell cycle stage, detailed immunophenotypes, apoptotic states, activation of signaling pathways, and others [reviewed by (99)]. This technique has thus been paramount in characterizing cell types, revealing the existence of previously unrecognized cell subsets, and for the isolation of functionally distinct cell subsets for the characterization of tumors. However, the design of multiparameter flow cytometry antibody panels is a challenging and laborious task, and most flow cytometry studies have so far focused on the in-depth analysis of specific cellular lineages, instead of a broad and system-wide approach.

In 2009, the advent of a new cytometry methodology, mass cytometry (CyTOF, cytometry by time-of-flight), overcame the limitation of spectral overlap by using metal-isotope-conjugated antibodies to detect antigens (100). The metal isotopes attached to each cell are distinguished by mass and quantified in a quadrupole time-of-flight mass spectrometer. A mass cytometer is theoretically capable of detecting over 100 parameters per cell, but current chemical methods limit its use to ~40–50 parameters, simultaneously. Mass cytometry has expanded the breadth of single-cell data in each experiment, making it highly suitable for systems-level analyses such as immunophenotyping of cancer microenvironments. By allowing the examination of large datasets at single-cell resolution, mass cytometry can be applied for the discovery of novel cell subsets as well as for the detection and identification of rare cells. Further advantages of mass cytometry are the irrelevance of autofluorescence, the low biological background as heavy metals are not naturally present in biological systems, and limited signal spillover between heavy metals, thereby reducing the complexity of panel design. Conversely, as compared to flow cytometry, mass cytometry suffers from a higher cell loss during acquisition, is more expensive, and is low-throughput, with a flow rate of up to 500 cells *per sec* as compared to thousands of cells *per sec* in flow cytometry. In addition, cells cannot be sorted for further analysis and forward- and side-scattered light is not detected.

Several studies have applied mass cytometry to further characterize immune cell profiles in peripheral blood or tissues from patients with breast cancer (101), renal cancer (102), melanoma (55, 56, 103–105), lung cancer (52, 106, 107), glioma (49, 50), colorectal cancer (57, 106, 108, 109), liver cancer (61, 110), ovarian cancer (111), and myeloma (112–115), among others. In addition to characterizing immune cell profiles of different tissue types, mass cytometry has also been used to characterize immunophenotypes in tumors and monitor changes during immunotherapy (56, 103–105, 114). In this way, factors that influence response to immunotherapy can be investigated and mechanisms at play during treatment can be characterized. This information can be used to understand and facilitate the identification and classification of responder *vs*. non-responders to cancer immunotherapy. Most of the studies so far have focused on the CTLA-4 and PD-1/PD-L1 axis of cancer immunotherapy, but novel immunotherapeutic targets such as co-inhibitory molecules LAG-3 or TIM-3 or co-stimulatory molecules such as OX40 and GITR are currently being explored in mice models and clinical trials (116). Moreover, mass cytometry has been employed to study antigen-specific T cells with a multiplex MHC class I tetramer staining approach, which has led to the identification of phenotypes associated with tumor antigen-specific T cells (106). Most studies applied mass cytometry for measuring cell surface or intracellular markers, but it can also be used to evaluate cell signaling processes relying on the analysis of protein phosphorylation (117). Altogether, these studies showed that immune responses in cancer are extremely diverse, within tumors from individual patients as well as between patients with equivalent tumor types. Hence, finding clinically-relevant characteristics based on overall differences can be challenging because of inter-patient variability; differences between cancer patients can be so large that they compromise the discovery of biomarkers.

Because the number of potential phenotypes (resulting from the combination of different markers) increases exponentially with the rise in number of antibodies being measured simultaneously, computational tools for the analysis and visualization of multidimensional data have become key in this field. Traditional workflows for analyzing flow cytometry datasets by manual gating are not efficient to capture the phenotypic differences in mass cytometry and complex flow cytometry data and suffer from individual user bias. In addition, flow and mass cytometry datasets can easily contain millions of cells, illustrating the need for scalable clustering algorithms for efficient analysis. Current single-cell computational tools employed for complex flow cytometry and mass cytometry datasets include unsupervised clustering-based algorithms such as SPADE (118), Phenograph (119), and FlowSOM (120). However, these clustering-based tools do not provide single-cell resolution of the data. On the other hand, non-linear dimensionality reduction-based algorithms such as t-SNE (121) are widely used tools but limited by the number of cells that they can analyze simultaneously, resulting in down-sampled datasets and non-classified cells. Recently, a hierarchical approach of the t-SNE dimensionality-reduction-based technique, HSNE, was described to be scalable to tens of millions of cells (122, 123). In addition, a novel algorithm has recently been implemented in the single-cell analysis field as a dimensionality reduction tool, called uniform manifold approximation and projection (UMAP) (124).

### Spatially-Resolved Data

Most of the multidimensional single-cell techniques such as flow cytometry, mass cytometry, and scRNA-seq require cellular dissociation to obtain cell suspensions prior to measuring the individual cells. Different dissociation methods are used, both mechanical and enzymatic, and may result in the loss of certain cell types and affect the expression of specific cell surface markers. Moreover, tissue specimens are often contaminated with blood or other tissues that are processed along with the tissue of interest. As such, not all subsets identified in single-cell data may be representative of the sample of interest. Another key limitation is the lack of information on spatial localization and cellular interactions within a tissue. Analysis of tissue sections by traditional IHC- and immunofluorescence-based methods are useful in providing spatial information (125), but are severely limited in the number of markers that can be measured simultaneously. Recent technological advances have greatly expanded the number of markers that can be captured on tissue slides. For instance, by applying the principles of secondary ion mass spectrometry to image antibodies conjugated to heavy metal isotopes in tissue sections with imaging mass cytometry (IMC) (126) and multiplexed ion beam imaging by time-of-flight (MIBI-TOF) (127). In both imaging approaches, conventional IHC workflows are used but with metal-isotope-conjugated antibodies that are detected through a time-of-flight mass spectrometer. In IMC, a pulsed laser is used to ablate a tissue section by rasterizing over a selected region of interest. The liberated antibody-bound ions are subsequently introduced into the inductively coupled plasma time-of-flight mass spectrometer. IMC can currently image up to 40 proteins with a subcellular resolution of 1 μm. The principle of MIBI-TOF is similar, but it makes use of a time-of-flight mass spectrometer equipped with a duoplasmatron primary oxygen ion beam rather than a laser. It currently enables simultaneous imaging of 36 proteins at resolutions down to 260 nm (128). Both techniques are, however, low-throughput due to the relatively long imaging time of 2 h per field of 1 mm^2^ in IMC and 1 h 12 min per field of 1 mm^2^ in MIBI-TOF (129). IMC has been applied to study tumor heterogeneity in several types of cancers, such as pancreatic cancer (130), biliary tract cancer (131), breast cancer (126, 132, 133), and colorectal cancer (108, 134). MIBI-TOF has been used to study the tumor-immune microenvironment of breast cancer (127, 128, 135, 136) and the metabolic state of T cells in colorectal cancer (109). These spatially-resolved, single-cell analyses have great potential to characterize the spatial inter- and intratumoral phenotypic heterogeneity, which can guide cancer diagnosis, prognosis and the selection of treatment. A recent study was able to extend IMC data by integration with genomic characterization of breast tumors and could, in this way, investigate the effect of genomic alterations on multidimensional tumor phenotypes of breast cancer (137).

Other multiplexed imaging techniques such as the Digital Spatial Profiling (DSP) system from NanoString and co-detection by indexing (CODEX) make use of DNA oligonucleotides. In DSP, antibodies or probes are tagged with unique ultraviolet-photocleavable DNA oligos that are released after ultraviolet exposure in specific ROIs and quantified (138). It enables simultaneous detection of up to 40 proteins or over 90 RNA targets from a tissue section and theoretically allows unlimited multiplexing using the NGS readout, but is time-consuming, does not allow for a reconstruction of the image, and has a lower resolution (10 μm) (129). In CODEX, antibodies conjugated to unique oligonucleotide sequences are detected in a cyclic manner by sequential primer extension with fluorescent dye-labeled nucleotides. CODEX currently allows the detection of over 50 markers with an automated fluidic setup platform including a three-color fluorescence microscope (139). Of note, throughput is limited by sequential detection of antibody binding. A disadvantage of CODEX, but also of IMC, is the lack of signal amplification which hampers the detection of lowly abundant antigens. A novel imaging technique, called Immuno-SABER, overcame this limitation by implementing a signal amplification step using primer exchange reactions. Immuno-SABER makes use of multiple DNA-barcoded primary antibodies that are hybridized to orthogonal single-stranded DNA concatemers, generated via primer exchange reactions (140). These primer exchange reactions allow multiplexed signal amplification with rapid exchange cycles of fluorophore-bearing imager strands. The Nanostring DSP platform has been used to study the tumor microenvironment and the outcome of various clinical trials of combination therapy for melanoma (141–144), interactions between macrophages and lymphocytes in metastatic uveal melanoma (145), immune cell subsets in lung cancer (129, 143), and tumor microenvironments of different metastases in prostate cancer (146). CODEX has been applied to study the immune tumor microenvironment of colorectal cancer patients with 56 protein markers simultaneously (147).

These multiplexed imaging techniques can be applied to snap-frozen as well as FFPE samples that are usually stored in clinical repositories. However, they raise new analysis challenges such as the visualization of 40 markers simultaneously, the image segmentation for cell determination, and algorithms for image-based expression profiles. To understand the tissue architecture, it is necessary to have prior knowledge on which cell types can be present and what their physical relationship to one another could be. Several computational approaches have been developed to enable data analysis of spatially-resolved multiplexed tissue measurements including HistoCAT (148) and ImaCytE (149). These approaches apply cell segmentation masks [using a combination of Ilastik (150) and CellProfiler (151)] to extract single-cell data from each image, which allow for deep characterization using multidimensional reduction tools such as t-SNE combined with the assessment of spatial localization and cellular interactions. In addition to cell-based analysis such imaging technologies also allow the employment of pixel-based analysis that do not depend on cell segmentation.

Integration of single cell transcriptome profiles with their spatial position in tissue context can be achieved by labeling of DNA, RNA, or probes using *in situ* hybridization (ISH). Traditional ISH techniques have been improved to allow the detection of single molecules, named single-molecule fluorescence ISH (smFISH) that can be used to quantitate RNA transcripts at single-cell resolution within a tissue context (152, 153). However, only a small number of genes can be measured simultaneously and a main limitation is the lack of cellular resolution to hundreds of micrometers. To improve the throughput, several highly multiplex methods of *in situ* RNA visualization have been developed such as osmFISH (154), sequential FISH [seqFISH (155) and seqFISH+ (156)] and error-robust FISH [MERFISH (157)]. These allow the subcellular detection of 100–10,000 transcripts simultaneously in single cells *in situ* by using sequential rounds of hybridization with temporal barcodes for each transcript, but require a high number of probes and are time-consuming. Furthermore, ISH can suffer from probe-specific noise due to sequence specificity and background binding. Another approach which may be more applicable for tumors is *in situ* RNA sequencing on tissue sections. STARmap (158) and FISSEQ (159) can profile a few hundreds to thousands of transcripts by using enzymatic amplification methods, but at lower resolution and sensitivity compared to seqFISH and MERFISH. Spatial Transcriptomics (160) and Slide-seq (161) can profile whole transcriptomes by using spatially barcoded oligo-dT microarrays. The spatial transcriptomics method has been used to study and visualize the distribution of mRNAs within tissue sections of breast cancer (160, 162), metastatic melanoma (56, 163), prostate cancer (164), and pancreatic cancer (165). These studies highlight the potential of gene expression profiling of cancer tissue sections to reveal the complex transcriptional landscape in its spatial context to gain insight into tumor progression and therapy outcome.

## Integration of Transcriptomic, (EPI)Genomic, Proteomic, and Spatially-Resolved Single-Cell Data

**Figure 2:**
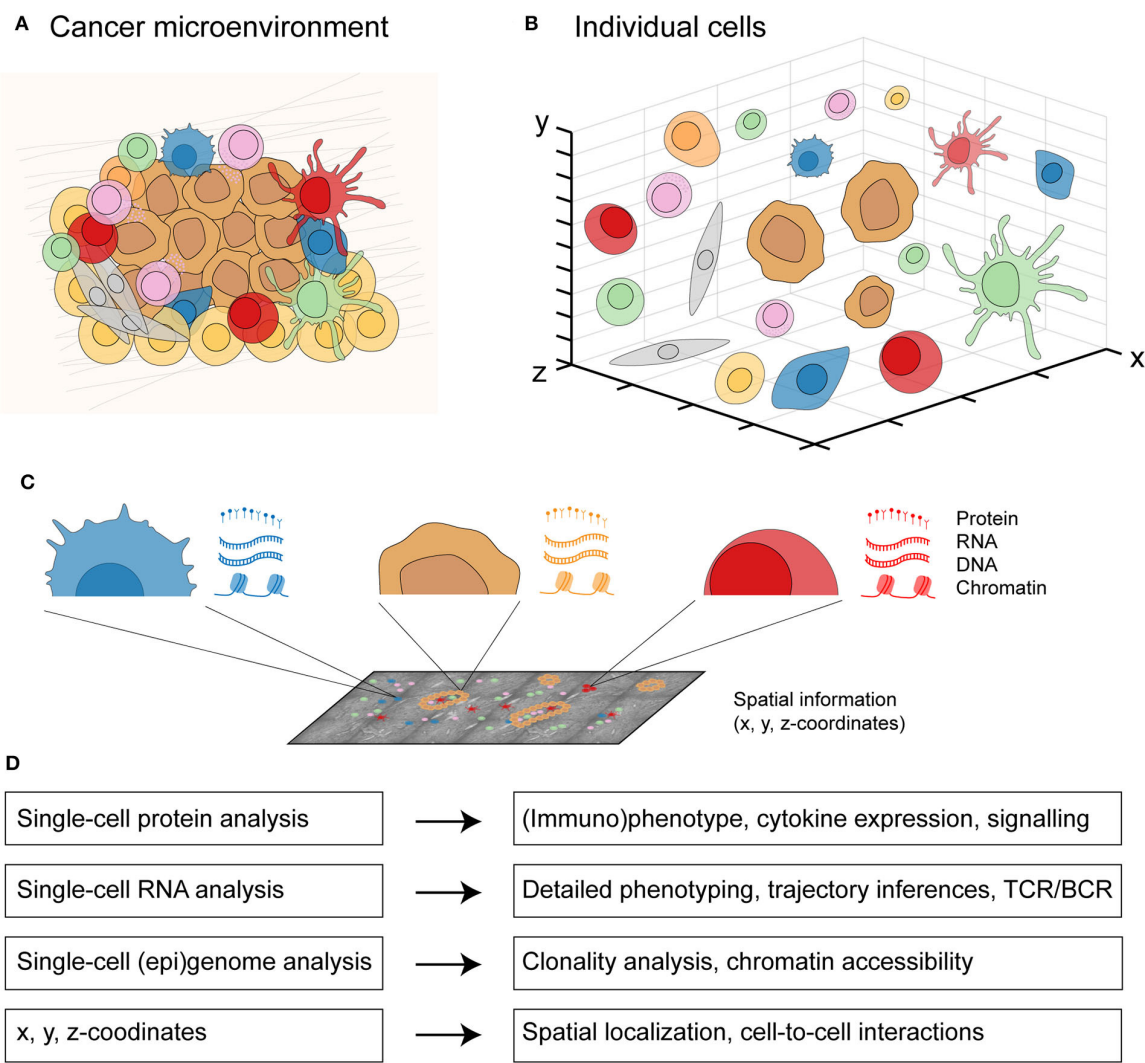
An integrated multicellular model of cancer. **(A)** From cells in a spatially-organized cancer microenvironment to **(B)** a three-dimensional view of individual cells. **(C)** From each individual cell in the cancer microenvironment, protein expression can be measured by single-cell protein analysis, RNA expression by single-cell RNA analysis, DNA and chromatin expression by single-cell (epi)genome analysis, and the x, y, z-coordinates with spatially-resolved analysis. **(D)** Integrating all four molecular layers for each cell will allow a detailed profiling from individual cell-to-cell interactions to whole tissue context.

Traditionally, each type of single-cell data has been considered independently to investigate a biological system. However, cancer is a spatially-organized system composed of many distinct cell types (Figure 2A). These different cell types including immune cells, stromal cells, and malignant cells can be visualized and investigated in an interactive manner (Figure 2B). By applying multi-omics to individual cells in the cancer microenvironment, the molecular landscape of every cell (44) can be defined with its proteome (proteins), transcriptome (RNA sequence), genome (DNA sequence), epigenome (DNA methylation, chromatin accessibility), and spatial localization (x, y, z-coordinates) (Figure 2C). Integrating these different molecular layers for each cell will allow a detailed profiling of cancer as a complex biological system (Figure 2D). Data integration approaches have classically been categorized in three groups: early (concatenation-based), intermediate (transformation-based), and late (model-based) stage integration (166). Early or intermediate stage integration approaches are more powerful than late stage integration since they can capture interactions between different molecular data-types. However, such approaches are also more challenging methodologically given the different data distributions across data types.

A number of studies have used complementary forms of multidimensional analysis on the same sample type in the context of cancer. We have performed a search strategy in PubMed, Web of Science, and Embase databases to find studies that have used mass cytometry in concert with scRNA-seq in the context of human cancer (Supplementary Table 1). An overview of the eight relevant studies that applied mass cytometry together with scRNA-seq to study human cancer and their integration stage is shown in Table 1. In addition, we performed a search strategy in PubMed, Web of Science, and Embase databases on studies that applied single-cell mass cytometry in concert with spatially-resolved data obtained by IMC or MIBI-TOF in human cancer (Supplementary Table 1). An overview of the two relevant studies and their integration stage is shown in Table 2. Notably, all different multidimensional datasets in these studies were analyzed separately and follow a late (model-based) stage integration. Only Goveia and colleagues applied an integration of clustered mass cytometry and scRNA-seq data (107). They merged scaled average gene expression data for each scRNA-seq cluster with scaled average protein expression data for each CyTOF cluster, an approach based on a recently described method from Giordani et al. (167). As they integrated the data only after clustering each modality separately, it is still considered late stage integration.

**Table 1:** Overview of studies applying mass cytometry together with single-cell RNA-sequencing to study human cancer heterogeneity.

**References**	**Methods for single-cell profiling**	**Cancer type**	**Integration stage**
Lavin et al. 2017 (52)	Mass cytometry and scRNA-seq	Lung cancer	Late
De Vries et al. 2019 (57)	Mass cytometry and scRNA-seq	Colorectal cancer	Late
Zhang et al. 2019 (61)	Mass cytometry and scRNA-seq	Liver cancer	Late
Sankowski et al. 2019 (49)	Mass cytometry and scRNA-seq	Glioma	Late
Halaby et al. 2019 (55)	Mass cytometry and scRNA-seq	Melanoma	Late
Goswami et al. 2020 (50)	Mass cytometry and scRNA-seq	Glioblastoma	Late
Goveia et al. 2020 (107)	Mass cytometry and scRNA-seq	Lung cancer	Late
Helmink et al. 2020 (56)	Mass cytometry and scRNA-seq	Melanoma	Late

*scRNA-seq, single-cell RNA-sequencing*.

**Table 2:** Overview of studies applying mass cytometry together with imaging mass cytometry or MIBI-TOF to study human cancer heterogeneity.

**References**	**Methods for single-cell profiling**	**Cancer type**	**Integration stage**
Zhang et al. 2019 (108)	Mass cytometry and IMC	Colon cancer	Late
Hartmann et al. 2020 (109)	Mass cytometry and MIBI-TOF	Colorectal cancer	Late

*IMC, imaging mass cytometry; MIBI-TOF, multiplexed ion beam imaging by time-of-flight*.

Integrating multiple single-cell datasets is a challenging task because of the inherently high levels of noise and the large amount of missing data. Furthermore, the ever-expanding scale of single-cell experiments to millions of cells poses additional challenges. Several methods have been proposed to integrate multimodal single-cell data. State-of-the-art methods focus on embedding both spatial and standard datasets into a latent space using dimensionality reduction, such as Seurat (16), LIGER (17), and Harmony (168), or by employing factor analysis, such as MOFA (169), MOFA+ (170), scMerge (171), and scCoGAPS (172). In addition, a recent study introduced gimVI as a model for integrating spatial transcriptomics data with scRNA-seq data to impute missing gene expression measurements (15). Of note, most of the methods so far follow an intermediate or late integration approach (166). As such, these methods overcome challenges due to the different data distributions across data types, but they are less powerful in capturing interactions between different molecular data types.

Several methodologies have been developed to simultaneously acquire multiple measurements from the same cell (Box 1). Although obtaining simultaneous measurements from the same cell is becoming more feasible, it is still more common to perform subsequent measurements from the same sample (different sets of cells). Integrating spatial-based assays with mRNA or protein expression measurements can be beneficial for several reasons. For instance, spatial measurements are often limited in terms of the number of features they can assess simultaneously, although the latest generations of MERFISH and seqFISH(+) can measure around 10,000 transcripts per cell. By integrating these imaging techniques with scRNA-seq, the amount of biologically-relevant information can be enhanced. Moncada et al. presented an integration of scRNA-seq with the spatial transcriptomics method generated from the same sample to study pancreatic cancer (165). A clear challenge when integrating spatial protein (e.g., IMC, MIBI-TOF, CODEX) with scRNA-seq data is the need to model relationships between mRNA and protein expression levels, thus adding an extra layer of complexity. The advent of CITE-seq, combining antibody-based detection of protein markers with transcriptome profiling, could be used to bridge this gap since it allows simultaneous measurement of both mRNA and surface protein marker expression. We foresee an important role for CITE-seq data in the integration of IMC, MIBI-TOF, and CODEX spatial data with scRNA-seq data. Recently, the integration of CITE-seq with CODEX as well as with IMC has been pioneered by Govek et al. (173).

Box 1 **Methods for the integration of transcriptomic, (epi)genomic, and proteomic single-cell data.**

The analysis of protein expression has been extended to include transcript measurements at the single-cell level. CITE-seq (69), REAP-seq (70), and PLAYR (180) can be used to detect mRNA and protein levels simultaneously in single-cells. In CITE-seq and REAP-seq, oligonucleotide-labeled antibodies are used to integrate cellular protein and transcriptome measurements. In PLAYR, mass spectrometry is used to simultaneously analyze the transcriptome and protein expression levels. The analysis of mRNA expression and methylation status in single cells can be achieved by scM&Tseq (181). In addition, mRNA expression and chromatin accessibility of single cells can be analyzed by sci-CAR (182), SNARE-seq (183), and Paired-seq (184). Chromatin organization and DNA methylation from a single nucleus can jointly be profiled by snm3C-seq (185). DR-seq (186) and G&T-seq (187) can assay genomic DNA and mRNA expression simultaneously in single cells, allowing correlations between genomic aberrations and transcriptional levels. Moreover, recent studies have reported on the development of single-cell triple-omics sequencing techniques, such as scTrio-seq (188) and scNMT-seq (189). In scTrio-seq, the transcriptome, genome, and DNA methylome of individual cells are jointly captured. Lastly, scNMT-seq jointly profiles transcription, DNA methylation, and chromatin accessibility, allowing for a thorough investigation of different epigenomic layers with transcriptional status.

## Potential Avenues of How the Integrated Data Will Help to Shed Light on the Complex Role of the Microenvironment in Cancer

Cancer heterogeneity has long been recognized as a factor complicating the study and treatment of cancer but, until recently, it was difficult to account for in cancer research. The advent of multidimensional single-cell technologies has shed light on the tremendous cellular diversity that exists in cancer tissues and heterogeneity across patients. Moving forward, it will be important to work on the integration of available (spectral) flow cytometry, mass cytometry, scRNA-seq, and spatially-resolved datasets to investigate commonalities and differences in cellular landscapes between cancer tissues. Multiple flow and mass cytometry datasets can be matched if they include a shared marker set between panels, thereby extending the number of markers per cell and allowing meta-analysis of different mass cytometry datasets with a common core of markers (174). In addition, cell-type references from different single-cell datasets can improve the functional characterization of cells (175). Such a system-wide approach will improve insights into how different components of the cancer microenvironment interact in a tissue context. This requires an extensive collaboration between multi-disciplinary research fields such as oncology, immunology, pathology, and bioinformatics.

Nevertheless, the development and widespread use of innovative methodologies also implies the development of analytical tools for the interpretation of complex datasets and their standardization across laboratories. Furthermore, systems-level analyses challenge a researcher's capacity to reconnect findings to their biological relevance. Studies should focus on the removal of unwanted variation and experimental noise in high-throughput single-cell technologies as well as the development of cell-type references, such as the Human Cell Atlas (176) and the Allen Brain Atlas (177) principles. We need to further develop algorithms to integrate data from different imaging and non-imaging single-cell technologies. Alternatively, technological developments should allow the acquisition of molecular profiles from single cells without the need of dissociating them from their tissue context. Lastly, it would be of great value to correlate multi-omics techniques with cell-to-cell signaling networks such as CellPhoneDB (178) and NicheNet (179). We expect that this integrated and comprehensive data can be used to create a multicellular model of cancer, from single cells to its tissue context, to understand and exploit cancer heterogeneity for improved precision medicine for cancer patients.

How will such system-wide approaches contribute toward more effective therapies for the treatment of cancer? With the advent of targeted therapy and immunotherapy, remarkable advances have been made that changed the management of oncologic treatment for a significant number of patients. However, still only a minority of cancer patients benefit from these therapies, and resistance to treatment remains a major complication in the clinical management of advanced cancer patients. Integrated multi-omics data can help to improve our understanding of the variability in treatment response and resistance mechanisms. By linking detailed molecular and immunological profiles of cells in the cancer microenvironment with sensitivity to specific therapies, potential targets for cancer treatments and associated biomarkers can be identified. This would also support a rational selection of patients that are most likely to benefit from specific treatments. Furthermore, integrated multi-omics data has the potential to guide the development of alternative therapies, for instance through the identification of resistance mechanisms. We expect that such system-wide approaches, with technologies that include spatial information, will become standard methodologies in cancer research in the coming years.

## Author Contributions

NV and AM performed the bibliographic research for the manuscript and designed the figures. NV, AM, FK, and NM jointly wrote the manuscript. All authors contributed to the article and approved the submitted version.

## Conflict of Interest

The authors declare that the research was conducted in the absence of any commercial or financial relationships that could be construed as a potential conflict of interest. The handling editor declared a past co-authorship with one of the authors NM.
