# Supplementary material for: Unraveling the Complexity of the Cancer Microenvironment With Multidimensional Genomic and Cytometric Technologies
Source: Front Oncol. 2020 Jul 23;10:1254. doi: 10.3389/fonc.2020.01254 (PMC7390924; doi:10.3389/fonc.2020.01254)
Supplement: Supplementary file 1 [file Table_1.DOCX]

**Supplementary table 1. Literature search strategy for PubMed, Web of Science, and Embase databases conducted on 27-02-2020.**

| Database | Mass cytometry and single-cell RNA-sequencing in human cancer | Mass cytometry and IMC or MIBI-TOF* in human cancer |
| --- | --- | --- |
| PubMed  Total  Relevant | 22 references^a^  6 references | 110 references^d^  1 reference |
| Web of Science  Total  Relevant | 59 references^b^, of which 29 unique  1 unique reference | 25 references^e^, of which 7 unique  0 unique references |
| Embase  Total  Relevant | 48 references^c^, of which 29 unique  1 unique reference | 24 references^f^, of which 6 unique  0 unique references |

Abbreviations: IMC; imaging mass cytometry, MIBI-TOF; multiplexed ion beam imaging by time-of-flight

**For mass cytometry and MIBI-TOF in human cancer, we found 1 additional relevant article on bioRxiv.*

^a^ (("single cell rna sequencing"[tw] OR "single cell rna seq"[tw] OR (("Sequence Analysis, RNA"[Mesh] OR "rna sequencing"[tw] OR "rna seq"[tw] OR rna seq*[tw] OR rnaseq*[tw]) AND ("Single-Cell Analysis"[Mesh] OR "Single Cell"[tw] OR "Single Cells"[tw]))) AND ("mass cytometry"[tw] OR mass cytometr*[tw] OR "CyTOF"[tw] OR ("Flow Cytometry"[mesh] AND "Mass Spectrometry"[mesh]) OR (("Flow Cytometry"[mesh] OR cytometr*[tw]) AND ("Mass Spectrometry"[mesh] OR "mass"[tw]))) AND ("Neoplasms"[mesh] OR neoplas*[tw] OR "tumor"[tw] OR "tumour"[tw] OR "tumors"[tw] OR "tumours"[tw] OR "cancer"[tw] OR "cancers"[tw] OR carcinoma*[tw] OR maligna*[tw] OR oncol*[tw] OR oncog*[tw] OR carcinog*[tw] OR metasta*[tw]))

^b^ TS=(("single cell rna sequencing" OR "single cell rna seq" OR (("RNA Sequence" OR "rna sequencing" OR "rna seq" OR "rna seq*" OR rnaseq*) AND ("Single Cell Analysis" OR "Single Cell" OR "Single Cells"))) AND ("mass cytometry" OR "mass cytometry" OR "mass cytometr*" OR "CyTOF" OR ("Flow Cytometry" AND "Mass Spectrometry") OR (("Cytometry" OR cytometr*) AND ("Mass Spectrometry" OR "mass")) OR "Flow Cytometr*") AND ("Neoplasm" OR neoplas* OR "tumor" OR "tumour" OR "tumors" OR "tumours" OR "cancer" OR "cancers" OR carcinoma* OR maligna* OR oncol* OR oncog* OR carcinog* OR metasta*)) NOT dt=(meeting abstract)

^c^ (("single cell rna sequencing".mp OR "single cell rna seq".mp OR (("RNA Sequence"/ OR "rna sequencing".mp OR "rna seq".mp OR "rna seq*".mp OR rnaseq*.mp) AND ("Single Cell Analysis"/ OR "Single Cell".mp OR "Single Cells".mp))) AND ("mass cytometry"/ OR "mass cytometry".mp OR "mass cytometr*".mp OR "CyTOF".mp OR ("Cytometry"/ AND "Mass Spectrometry"/) OR ((exp "Cytometry"/ OR cytometr*.mp) AND ("Mass Spectrometry"/ OR "mass".mp))) AND (exp "Neoplasm"/ OR neoplas*.mp OR "tumor".mp OR "tumour".mp OR "tumors".mp OR "tumours".mp OR "cancer".mp OR "cancers".mp OR carcinoma*.mp OR maligna*.mp OR oncol*.mp OR oncog*.mp OR carcinog*.mp OR metasta*.mp)) NOT (conference review or conference abstract).pt

^d^ (("mass cytometry"[tw] OR "CyTOF"[tw] OR ("Flow Cytometry"[majr] AND "Mass Spectrometry"[majr]) OR (("Flow Cytometry"[majr] OR cytometr*[tiab]) AND ("Mass Spectrometry"[majr] OR "mass"[tiab]))) AND ("imaging mass cytometry"[tw] OR ("imaging"[tw] OR "multiplexed ion beam imaging"[tw] AND ("mass cytometry"[tw] OR "CyTOF"[tw] OR ("Flow Cytometry"[majr] AND "Mass Spectrometry"[majr]) OR (("Flow Cytometry"[majr] OR cytometr*[tiab]) AND ("Mass Spectrometry"[majr] OR "mass"[tiab]))))) AND ("Neoplasms"[mesh] OR neoplas*[tw] OR "tumor"[tw] OR "tumour"[tw] OR "tumors"[tw] OR "tumours"[tw] OR "cancer"[tw] OR "cancers"[tw] OR carcinoma*[tw] OR maligna*[tw] OR oncol*[tw] OR oncog*[tw] OR carcinog*[tw] OR metasta*[tw])) AND ("2013/01/01"[PDAT] : "3000/12/31"[PDAT])

^e^ TS=(("mass cytometry" OR "mass cytometry" OR "CyTOF" OR (("Flow Cytometry" OR cytometr*) NEAR5 ("Mass Spectrometry" OR "mass"))) AND ("imaging mass cytometry" OR "multiplexed ion beam imaging" OR ("imaging" NEAR5 ("mass cytometry" OR "CyTOF"))) AND ("Neoplasm" OR neoplas* OR "tumor" OR "tumour" OR "tumors" OR "tumours" OR "cancer" OR "cancers" OR carcinoma* OR maligna* OR oncol* OR oncog* OR carcinog* OR metasta*)) AND py=(2013 OR 2014 OR 2015 OR 2016 OR 2017 OR 2018 OR 2019 OR 2020) NOT dt=(meeting abstract)

^f^ (("mass cytometry"/ OR "mass cytometry".ti,ab OR "CyTOF".ti,ab OR (*"Flow Cytometry"/ AND *"Mass Spectrometry"/) OR ((exp *"Flow Cytometry"/ OR cytometr*.ti,ab) AND (exp *"Mass Spectrometry"/ OR "mass".ti,ab))) AND ("imaging mass cytometry".ti,ab OR "multiplexed ion beam imaging".ti,ab OR ("imaging".ti,ab ADJ5 ("mass cytometry".ti,ab OR "CyTOF".ti,ab))) AND (exp "Neoplasm"/ OR neoplas*.mp OR "tumor".mp OR "tumour".mp OR "tumors".mp OR "tumours".mp OR "cancer".mp OR "cancers".mp OR carcinoma*.mp OR maligna*.mp OR oncol*.mp OR oncog*.mp OR carcinog*.mp OR metasta*.mp)) AND (2013 OR 2014 OR 2015 OR 2016 OR 2017 OR 2018 OR 2019 OR 2020).yr NOT (conference review or conference abstract).pt
